# Isolation and Genetic Characterization of Japanese Encephalitis Virus Two Decades after Its Elimination in Singapore

**DOI:** 10.3390/v14122662

**Published:** 2022-11-28

**Authors:** Ming Jie Lim, Zhi Yang Loh, Hui Ling Yeo, Surya Pavan Yenamandra, Marcella Kong, Hao Yang Pang, Meng Han Lee, Mahathir Humaidi, Cliff Chua, Jane Griffiths, Lee Ching Ng, Hapuarachchige Chanditha Hapuarachchi, Diyar Mailepessov

**Affiliations:** 1 Environmental Health Institute, National Environment Agency, Singapore 228231, Singapore; 2 School of Biological Sciences, Nanyang Technological University, Singapore 639798, Singapore

**Keywords:** Japanese encephalitis virus, JEV genotype I, phylogenetic analysis

## Abstract

Japanese encephalitis virus (JEV) is an important arbovirus in Asia that can cause serious neurological disease. JEV is transmitted by mosquitoes in an enzootic cycle involving porcine and avian reservoirs, in which humans are accidental, dead-end hosts. JEV is currently not endemic in Singapore, after pig farming was abolished in 1992; the last known human case was reported in 2005. However, due to its location along the East-Asian Australasian Flyway (EAAF), Singapore is vulnerable to JEV re-introduction from the endemic regions. Serological and genetic evidence in the last decade suggests JEV’s presence in the local fauna. In the present study, we report the genetic characterization and the first isolation of JEV from 3214 mosquito pools consisting of 41,843 *Culex* mosquitoes, which were trapped from April 2014 to May 2021. The findings demonstrated the presence of genotype I of JEV (n = 10), in contrast to the previous reports of the presence of genotype II of JEV in Singapore. The genetic analyses also suggested that JEV has entered Singapore on several occasions and has potentially established an enzootic cycle in the local fauna. These observations have important implications in the risk assessment and the control of Japanese encephalitis in non-endemic countries, such as Singapore, that are at risk for JEV transmission.

## 1. Introduction

Japanese encephalitis (JE) is the most significant vaccine-preventable neurological disease in Asia and the Western Pacific region [1,2]. The first confirmed cases of JE were detected in Japan in 1924, and it was subsequently reported in the rest of Asia, as well as in parts of Russia and Australia. At least 24 countries are currently considered to be at risk for JE transmission [1,3,4]. Based on 2011 estimates, the global annual caseload of JE was approximately 68,000 cases, with at least 13,000 deaths [5]. The global burden of JE is likely to increase as regions that were formerly less favorable to JE experience more hospitable conditions for its spread due to climate change [6,7,8]. This is most evident in the recent JE outbreak in Australia, with 37 locally-acquired cases reported; the outbreak occurred 20 years after the last known human case [9].

Japanese encephalitis virus (JEV), the causative agent of JE, is a positive-sense, single-stranded RNA virus of the Flaviviridae genus. JEV has five known genotypes (GI–GV) [10,11,12,13,14], including two sub-genotypes of GI—GI-a and GI-b [15]. The JEV genome is approximately 11 kilobases long and encodes ten proteins in an open reading frame flanked by two non-coding regions (NCRs) at the 5′ and 3′ ends. The ten proteins that are encoded are three structural proteins (capsid, membrane, and envelope) and seven non-structural (NS) proteins (NS1, NS2A, NS2B, NS3, NS4A, NS4B, and NS5) [16,17].

JEV is transmitted by *Culex* mosquitoes; *Culex tritaeniorhynchus* is the primary vector [18,19], and other *Culex* mosquitoes are secondary vectors. Other genera of mosquitoes, including *Aedes*, *Anopheles*, and *Mansonia,* may act as regional or potential secondary vectors (reviewed by Pearce, et al. [20]). The main reservoir hosts of JEV are birds, primarily of the family *Ardeidae*, while swine act as amplifying hosts [1,21]. Humans are incidental, dead-end hosts [2,22]. Before 2000, GIII was the most prominent JEV genotype in all endemic areas, but it is currently being supplanted by GI in Asia [23,24,25].

Prior to the abolishment of pig farming in 1992, Singapore had an average of 14 JE cases annually, but has had almost no cases since then [26,27]. However, the country has been identified as one of the 24 countries with a JE transmission risk [3,4]. A highly-urbanized island city-state, Singapore nevertheless retains much of its greenery and biodiversity through Nature Reserves (NRs), forest parks and urban parks, and mangroves [28]. Its tropical climate, combined with its varied forest and urban habitats, provide a conducive environment for at least 180 species of mosquitoes, including *Cx. tritaeniorhynchus* [29]. The country is also home to wild boars [30,31,32] and local wild birds. Additionally, migratory birds stopover in Singapore, which is located along the East-Asian Australasian Flyway (EAAF) [33]. Ardeid birds are present in both the local (e.g., Grey Herons and Yellow Bitterns) and the migratory bird populations (e.g., Little Egrets, Chinese Pond Herons, and Black Bitterns) [34].

A serological study that was carried out from 2010 to 2018, as well as an investigation of serum samples that were collected between 1996 and 1999, indicated JEV exposure in the wild boar and the wild bird populations in Singapore [26,35], suggesting that JEV may be enzootic in Singapore. Entomology-based surveillance that was initiated in 2011 detected five JEV-positive mosquito pools at a local Avian Sanctuary, further supporting this notion. The JEV sequences that were derived from those mosquito pools clustered with JEV GII isolates detected in Indonesia [36]. The seroconversion of sentinel chickens during surveillance carried out from 2013 to 2014 further suggested active JEV transmission in the rural areas of Singapore [35]. Although JEV had previously been detected in mosquito vectors in Singapore [36,37], virus isolation had not been successful [36]. In the present study, we report the first isolation and detection of JEV GI from *Culex* mosquitoes in Singapore. This study also describes the genetic characteristics of the newly detected JEV strains. The findings suggest that JEV has potentially established enzootic transmission locally, which has important implications on the risk assessment and vector control policies in Singapore.

## 2. Materials and Methods

### 2.1. Entomological and Virological Surveillance

**Figure 1:**
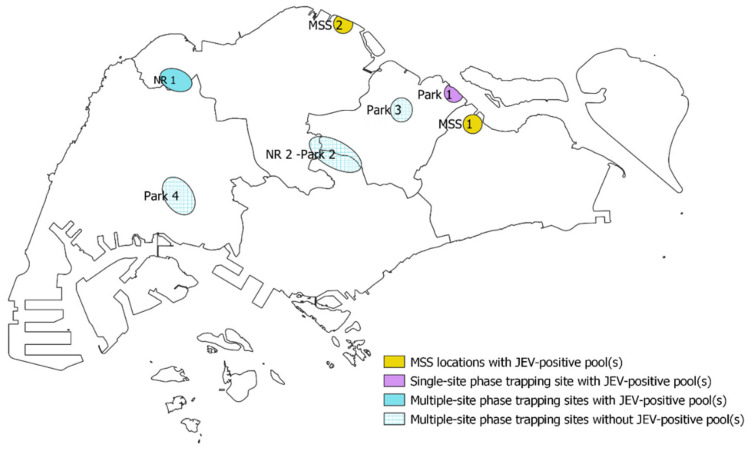
Locations of the five sites used in JEV entomological surveillance in this study, as well as the two locations where JEV was detected in mosquitoes that were obtained from MSS. The single-site study site is in purple and the multi-site study sites are in light blue. MSS locations are in yellow. The solid shapes indicate areas where JEV-positive pools were observed, while the hatched shapes indicate areas where JEV-positive pools were not observed. See Figure S1 for the approximate locations where ardeid birds and wild boars were sighted in Singapore.

Entomological surveillance and virological surveillance of JEV was carried out near-monthly; this occurred in two phases—a single-site phase from April 2014 to August 2016, and a multiple-site phase from September 2019 to May 2021. During the single-site phase, one surveillance site was set up at Park 1, which was in the north-eastern region of Singapore. During the multiple-site phase, four surveillance sites were set up at NR 1, NR 2-Park 2, Park 3, and Park 4 (Figure 1). NR 2 and Park 2 were considered as one site due to their proximity to each other. These study sites were selected based on their proximity to slow-moving or still water bodies and the available information on the presence of JEV hosts, such as ardeid birds and wild boars, and the presence of JEV vectors. These selection criteria were adopted to maximize the possibility of capturing JEV-positive mosquitoes because of the lack of evidence on active transmission of JEV in Singapore.

Mosquitoes were trapped over two consecutive nights during the single-site phase, and for 18–20 h during the multiple-site phase. Each trapping session was conducted overnight, including crepuscular periods, using Centers for Disease Control (CDC) light traps baited with dry ice and incandescent light. Four to nine locations were selected at each surveillance site, and traps were positioned approximately 1.5–2.0 m above ground in shaded areas adjacent to water bodies where possible. Traps were set between 15:00 and 17:00 h and were collected the next day between 09:00 and 11:00 h.

Additionally, *Culex* genus mosquitoes that were trapped from November 2020 to May 2021 as part of the malaria surveillance activities of the National Environment Agency (NEA) were also included in this study. The Malaria Surveillance Section (MSS) conducts on-going catches using the Night Catcher (a modified form of the CDC light trap [38]) in 45-day cycles at 116 different locations in Singapore. Each MSS trapping session was carried out over a 12-h period overnight, including crepuscular periods. The two MSS trapping locations where JEV-positive pools were observed are indicated as MSS 1 and MSS 2 (Figure 1).

### 2.2. Mosquito Identification

Mosquito specimens were transported on dry ice to the Environmental Health Institute (EHI), where they were sorted using mosquito identification keys [39,40,41,42,43,44,45]. For the single-site phase, mosquito specimens were identified to species level where possible. For the multiple-site phase, mosquito specimens were initially identified to genus level, until the first detection of JEV, whereupon they were identified to species level where possible. In the case of morphologically similar species, the specimens were identified to the species group. Damaged specimens were identified to the genus level. For the start of the multiple-site phase, all female *Culex* mosquitoes were pooled by trapping location and trapping month in pools of up to 100 specimens. Otherwise, females from six *Culex* vector species—*Cx. bitaeniorhynchus*, *Cx. gelidus*, *Cx. quinquefasciatus*, *Cx. sitiens*, *Cx. tritaeniorhynchus*, and *Culex* spp. *vishnui* subgroup (excluding *Cx. tritaeniorhynchus*)—were pooled according to the species, trapping location, and trapping month, in pools of up to 100 specimens. All specimen pools were stored at −80 °C until further analysis.

### 2.3. RNA Extraction from Mosquito Specimens

Pooled mosquito samples were homogenized in 500 μL of universal transport media (Copan Diagnostics, Murrieta, CA, USA) using either the Mixer Mill MM 400 (Retsch Technology GmbH, Haan, Germany) or the 1600 MiniG homogeniser (Spex SamplePrep, Metuchen, NJ, USA), and centrifuged at 5000× *g* for 5 min. Viral RNA was extracted from the mosquitoes collected during the single-site phase using 140 μL of the homogenate and the QIAamp Viral RNA mini kit (Qiagen Group, Hilden, Germany), according to the manufacturer’s instructions. For mosquitoes collected during the multi-site phase, as well as the mosquitoes obtained from MSS, viral RNA was extracted from 200 µL of mosquito homogenate using the IndiMag Pathogen kit (Indical Bioscience GmbH, Leipzig, Germany) and the KingFisher Flex system (Thermo Fisher Scientific Inc., Waltham, MA, USA), as per manufacturer’s instructions. Eluted RNA and the remaining homogenates were stored at −80 °C until further use.

### 2.4. Detection of JEV by Reverse Transcriptase-PCR (RT-PCR)

Extracted RNA was screened for JEV, as described in Yap, Mailepessov, Lim, Chan, How, Humaidi, Yeo, Chong, Lam-Phua, Lee, Okumura, Vythilingam and Ng [36]. Briefly, positive RNA controls of JEV (Nakayama strain) were extracted from Vero cell (ATCC CCL-81; ATCC, Manassas, VA, USA) supernatants using a QIAmp Viral RNA Mini kit (Qiagen), as described above. JEV was detected by using a modified RT-PCR assay adopted from Scherret, et al. [46] and Santhosh, et al. [47]. All reactions were performed using the QuantiTect^®^ SYBR^®^ Green RT-PCR kit (Qiagen) in a final volume of 20 µL, with 5 µL of extracted RNA and 10 µM of each primer. The cycling conditions were as follows: reverse transcription at 50 °C for 20 min and inactivation at 95 °C for 15 min; 40 cycles at 94 °C for 15 s, 58 °C for 30 s, and 72 °C for 30 s; melting curve analysis was conducted from 65 to 95 °C, with a slope of 0.1 °C/s, for validation of the amplified products; cycling conditions concluded with a cooling step at 37 °C for 20 s.

### 2.5. Sequencing and Analysis of the Envelope Gene of JEV

The envelope (*E*) gene of JEV was amplified using two pairs of primers, as described in Schuh, et al. [48]. Complementary DNA was first synthesized using the Maxima H Minus First Strand cDNA synthesis kit (Thermo Fisher Scientific Inc.), and subsequent amplifications were performed using Phusion Flash High-Fidelity PCR Master Mix (Thermo Fisher Scientific Inc.), as per the manufacturer’s recommendations. The amplified products (1700 and 800 bp) were purified using the FavorPrep GEL/PCR Purification Kit (Favorgen, Ping Tung, Taiwan) before sequencing at a commercial sequencing facility using the BigDye terminator Cycle Sequencing Kit protocol (Applied Biosystems, Thermo Fisher Scientific Inc., Waltham, MA, USA).

Raw sequence data were analyzed as described in Yap, Mailepessov, Lim, Chan, How, Humaidi, Yeo, Chong, Lam-Phua, Lee, Okumura, Vythilingam and Ng [36]. A phylogenetic tree was constructed using 129 truncated (701 bp) JEV *E*-gene sequences; this included the newly detected JEV sequences in the present study (n = 10) and 119 JEV sequences that were retrieved from the NCBI GenBank database (Table S1). The sequences were aligned using MUSCLE in MEGA version X 10.2.5 [49], and a phylogenetic tree was constructed using the maximum likelihood (ML) method. The best fitting model, which was the Tamura-Nei model [50] with gamma-distributed substitution sites (TN93+G), was determined via the model selection tool in MEGA version X 10.2.5. The tree was constructed using TN93+G with 1000 bootstrap reiterations and rooted with the truncated (701 bp) *E*-gene from Murray Valley Encephalitis virus (MVEV; NCBI Reference Sequence no. NC_000943.1). Amino acid sequences were also obtained via MEGA version X 10.2.5. Sequence identity matrices were generated with the MUSCLE tool in EMBL-EBI with default parameters [51,52]. The newly generated *E*-gene sequences from this study were deposited in the GenBank nucleotide database (GenBank Accession Nos. KT346354, MW802631, MW802633 to MW802637, and ON804794 to ON804796).

### 2.6. JEV Isolation

Mosquito homogenates that were positive for JEV were filtered (Minisart 0.45 microns; Sartorius, Göttingen, Germany) before inoculation onto C6/36 cells (ATCC CRL-1660; ATCC) at 90% confluency. The C6/36 cells were incubated in Leibovitz L-15 media (Thermo Fisher Scientific Inc.) with 2% fetal bovine serum (FBS) (Thermo Fisher Scientific Inc.) at 33 °C and monitored for one week. Approximately 1/5 of cell culture suspension was then transferred to freshly prepared 90% confluent C6/36 cells, topped up with fresh 2% FBS Leibovitz L-15 media, and incubated again at 33 °C for one week. This step was repeated one more time for a maximum of three passages. If cytopathic effects or syncytia were observed, the cells were harvested and kept at −80 °C.

### 2.7. Sequencing and Phylogenetic Analysis of the Whole Genomes of JEV

Viral RNA was extracted from cell culture suspension using the QIAmp Viral RNA Mini kit (Qiagen), according to manufacturer’s instructions. Multiple amplicons covering the whole genome were then synthesized using the primers in Table S3 and a protocol similar to that of *E*-gene amplification, except with an annealing temperature of 60 °C. The amplification products were purified with the FavorPrep GEL/PCR purification kit (Favorgen) and sequenced at a commercial sequencing facility using the BigDye terminator Cycle Sequencing Kit protocol (Applied Biosystems, Thermo Fisher Scientific Inc.).

Raw sequence data were trimmed and assembled using SeqMan Pro 15.3.0 from the Lasergene software suite (DNAStar Inc., Madison, WI, USA) and BioEdit 7.2.5 to obtain consensus sequences. Where a consensus could not be reached for a sequence call in the contig, the position was represented by the appropriate IUPAC ambiguous base symbol.

A whole-genome phylogenetic tree was constructed using 50 JEV sequences; 3 sequences were generated during the present study and 47 sequences were obtained from the NCBI GenBank database (Table S4). The sequences were aligned in MEGA version X 10.2.5; as above, the best fit model was determined to be the Tamura-Nei model, but with invariant and gamma-distributed substitution sites (TN93+G+I). The tree was constructed using TN93+G+I with 1000 bootstrap iterations and rooted with MVEV (NCBI Reference Sequence no. NC_000943.1). The amino acid translation was carried out via the Translate tools from Expasy [53] and the Sequence Manipulation Suite [54]. Sequence identity matrices of whole genome data sets were also generated as above. The whole genome sequences that were generated during the present study were deposited in the GenBank nucleotide database (GenBank Accession Nos. ON804797 to ON804799).

### 2.8. Mutation Analyses

Amino acid mutations that were unique to the study sequences were determined by comparing them with all of the JEV GI complete coding sequences available in the NCBI database (n = 158) as of 03 November 2022. Genotype I sequences were selected by constructing a phylogenetic tree consisting of all complete coding sequences of JEV (n = 404) available in the NCBI database. A consensus sequence of the GI complete coding sequences (n = 158) was generated using the PAM40 matrix with the *cons* module from the EMBOSS suite [55].

## 3. Results

### 3.1. Detection of JEV in Field-Caught Mosquitoes

A total of 54,308 *Culex* mosquitoes collected with CDC light traps and Night Catcher traps were screened in 3261 pools (Table 1). JEV was detected by RT-PCR in five *Culex* spp. pools and five *Cx. tritaeniorhynchus* pools, and virus isolation was successful in three pools. JEV was not found in the other five *Culex* species/species group that were screened.

Of the seven JEV-positive pools that were collected with the CDC light traps, one was collected from the north-eastern Park 1 in 2014, and six were collected from the north-western NR 1 in 2019. Of the three positive pools that were collected with the Night Catcher traps, two were collected in the north-eastern MSS 1 in 2020, while one was collected in the northern MSS 2 in 2021 (Table 2).

### 3.2. Sequencing, Phylogenetic Analysis, and Genetic Characterization of Detected JEVs

**Figure 2:**
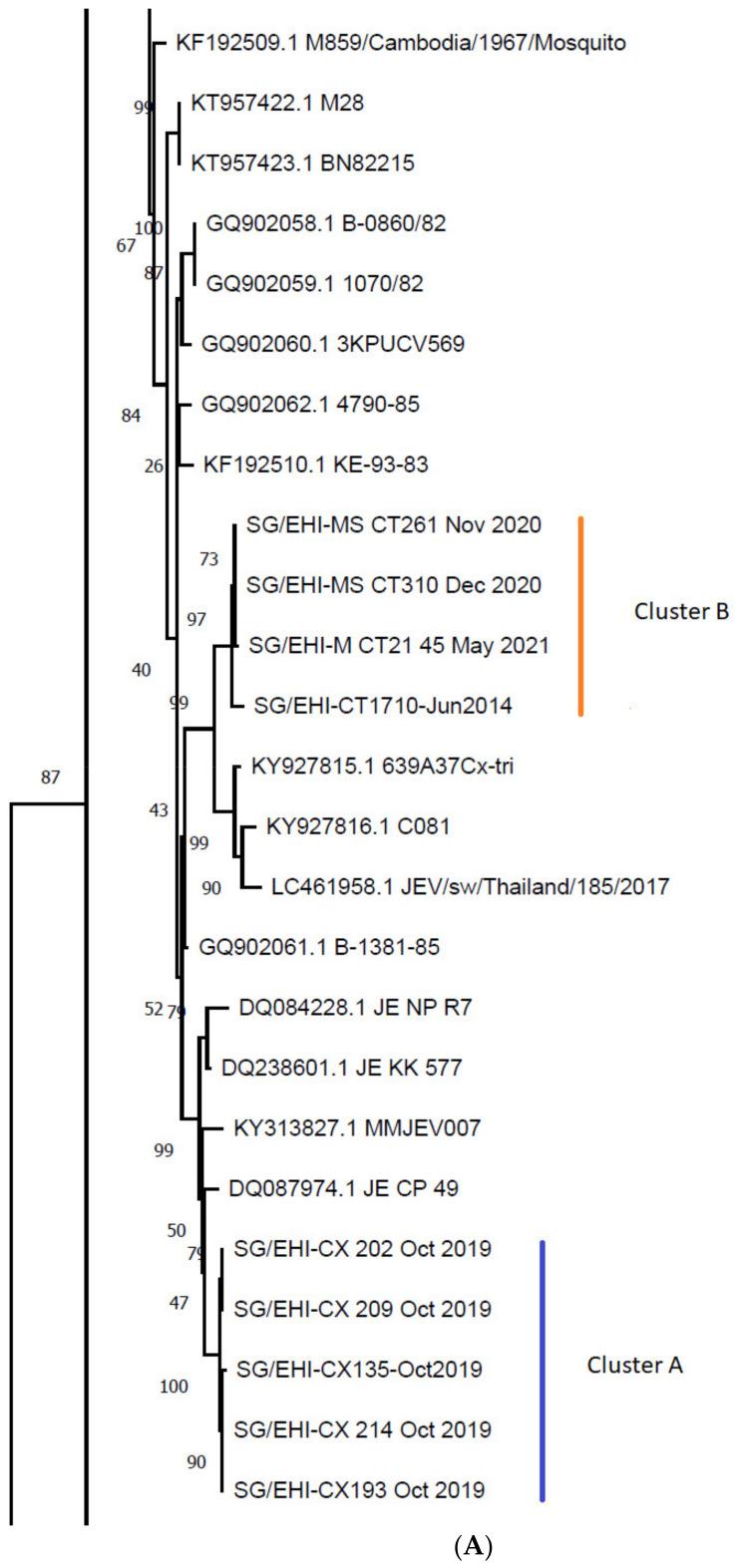

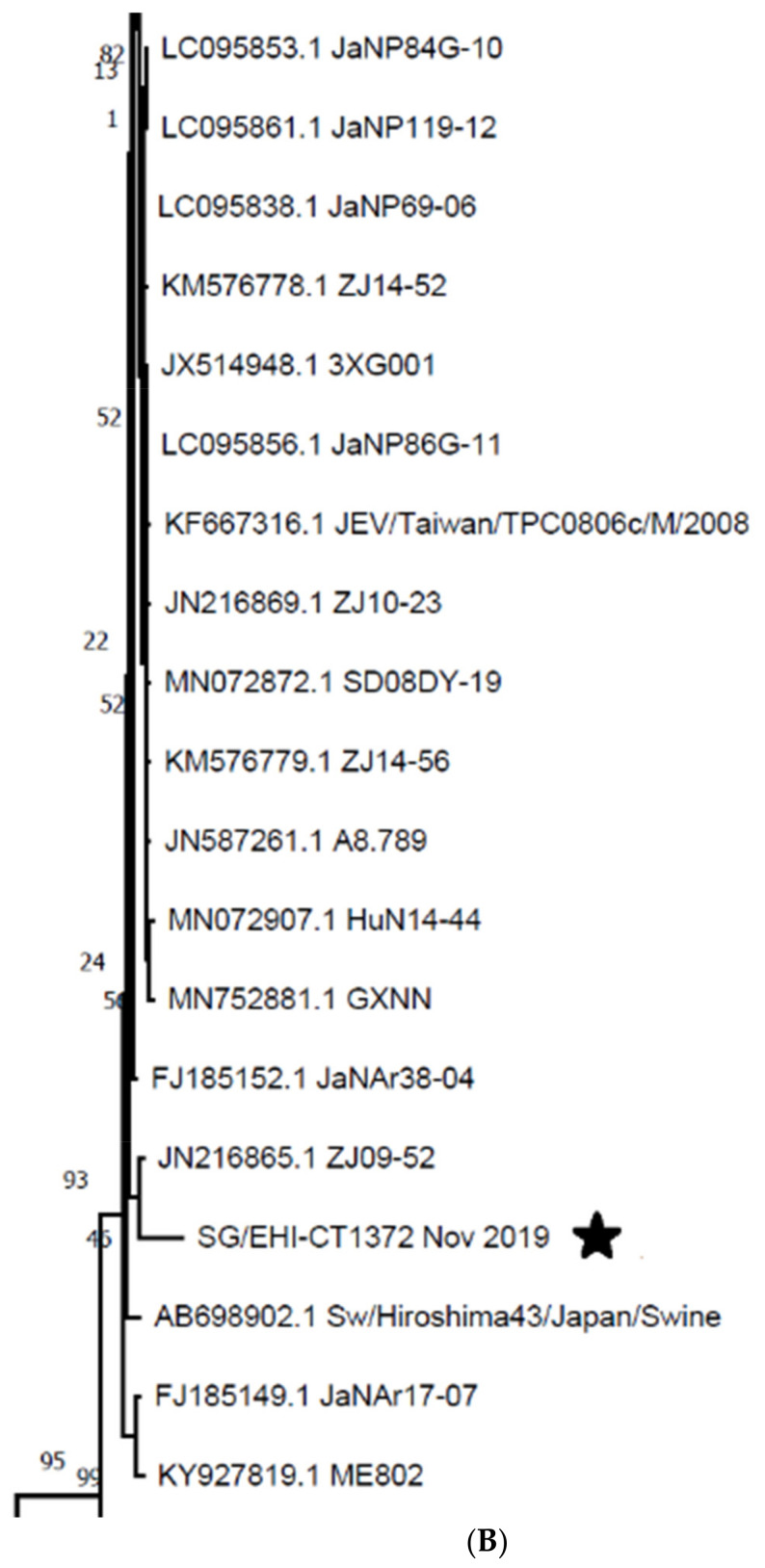
Maximum likelihood phylogenetic analysis of 129 truncated (701 bp) JEV *E*-gene sequences. This tree was constructed using TN93+G with 1000 bootstrap iterations; the outgroup is MVEV (NCBI Reference Sequence no. NC_000943.1). Only parts of the tree are presented for easier viewing (see Figure S1 for the full tree): (**A**) Sub-genotype I-a; (**B**) Partial sub-genotype I-b. The JEV *E*-gene sequences presented in this study are indicated with cluster A (blue) and cluster B (orange), as well as a star for SG/EHI-CT1372. Each node is labelled with the GenBank Accession No. (where available) followed by the sequence name.

Partial *E*-gene sequences of JEV (701 bp) were obtained from all ten of the positive pools. Direct PCR amplification of complete genome fragments using the RNA extracted from the JEV-positive mosquito pools was not successful. However, complete genome sequences were generated from three pools where JEV was successfully isolated. All newly-generated JEV *E*-gene sequences (n = 10) belonged to GI (Figure 2 and Figure S2). They shared 91.58–100% nucleotide and 95.71–100% amino acid similarity among them (Table 3). Nine *E*-gene sequences belonged to two distinct clades (clusters A and B) in sub-genotype GI-a, while the remaining sequence (SG/EHI-CT1372) belonged to GI-b (Figure 2 and Figure S1). SG/EHI-CT1372 clustered with ZJ-09-52 (GenBank Accession No. JN216865), which was reported from China in 2009. SG/EHI-CX135, -193, SG/EHI-CX 202, -209, and -214 formed a distinct cluster (cluster A) that was closely related to the isolate JE_CP_49 (GenBank Accession No. DQ087974) from Thailand in 2003. These five local sequences in cluster A demonstrated high (99.43–100%) nucleotide sequence identity and 100% amino acid sequence identity. SG/EHI-CT1710, -MS_CT310, -MS_CT261, and -M_CT21_45 formed another cluster (cluster B) that was most closely related to a cluster of sequences (639A37Cx-tri, C081, and JEV/sw/Thailand/185/2017; GenBank Accession Nos. KY927815-816, and LC461958) from Cambodia and Thailand in the 2010s. Cluster B sequences showed high nucleotide and amino acid similarity (99–100% and 100%, respectively) between one another, except for SG/EHI-M_CT21_45, which showed an amino acid similarity of 99.57%.

**Figure 3:**
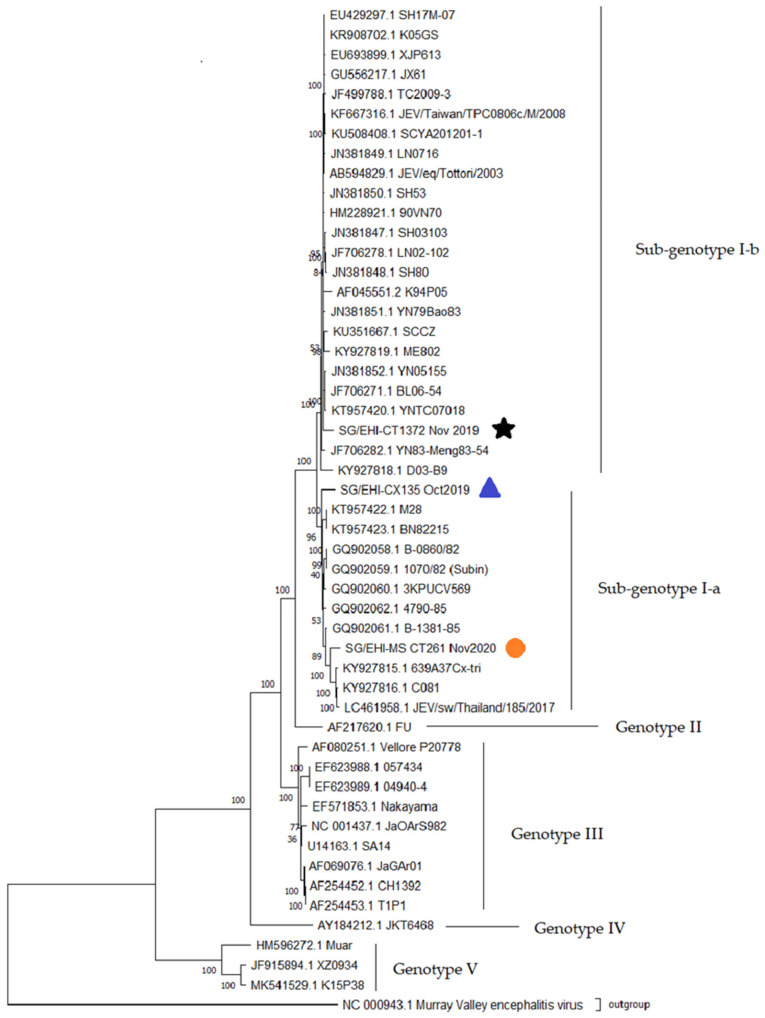
Maximum likelihood phylogenetic analysis of 50 JEV whole genome isolates. This tree was constructed using TN93+G+I with 1000 bootstrap iterations; the outgroup is MVEV (NCBI Reference Sequence no. NC_000943.1). The JEV isolates presented in this study are indicated with a star: SG/EHI-CT1372; blue triangle: SG/EHI-CX135; orange circle: SG/EHI-MS_CT261. The colors correspond to the clusters indicated in Figure 2. Genotypes are as indicated. Each node is labelled with the GenBank Accession No. (where available) followed by the isolate name.

Similar to the *E*-gene analysis, all three of the whole genomes that have been reported in the present study (SG/EHI-CT1372, -CX135, and -MS_CT261) belonged to GI. They shared 92.99–93.33% nucleotide and 94.39–96.75% amino acid similarity among them, demonstrating a clear genetic distance between one another (Table 4). SG/EHI-CT1372 was the most distant among the three isolates and belonged to GI-b (Figure 3). This isolate was most closely related to an isolate that was reported from China in 1983 (GenBank Accession No. JF706282). The remaining two sequences belonged to GI-a and clustered with JEV isolates that were reported from China (1977 & 1982), Thailand (1982, 1985, & 2014), Australia (2000–2004), and Cambodia (2014 & 2015) (GenBank Accession Nos. KT957422-423, GQ902058-061, KY927815-816, LC461958, and MT253732-734).

Among the 5′-NCRs of the three newly-detected whole genome sequences, there was only one mutation in SG/EHI-CT1372 (T > G) that was located in one of the loops of the first 5′-NCR stem loop structure (SLA). On the other hand, the 3′-NCRs had 28 mutations among the three isolates; 23 of these were in RNA secondary structures (Table S5). A total of 13 of the 23 mutations were positioned in stems of various 3′-NCR structures, while the remaining 10 mutations were in loop structures. A total of 5 of the 23 mutations belonged to significant regions in these structures, as described by Liu, et al. [56] (Table 5).

### 3.3. Amino Acid Differences between the New Singaporean JEV Sequences and the GI Consensus Sequence Derived from Published GI Sequences

A total of 30 unique amino acid differences were observed in the study sequences (n = 10) as compared to the JEV GI consensus sequence (Table 6 and Table 7). These mutations were novel among all of the JEV GI sequences (n = 158) that were reported in the NCBI database as of November 2022. Of these, 21 mutations were found in the non-structural proteins (Table 7). Of the remaining 9 mutations in the structural polyprotein, 7 substitutions were found in the E protein (Table 6). The mutation profiles of three complete genomes (SG/EHI-CT1372, SG/EHI-CX135, and SG/EHI-MS_CT261) were clearly distinguishable, indicating a clear genetic distinction among them. Notably, the unique mutation profile of SG/EHI-MS_CT261 consisted of more mutations in the non-structural polyprotein than the remaining two isolates. Among the 30 novel mutations, non-conservative substitutions occurred at 12 positions. Their distribution was proportionately higher in the structural polyprotein, suggesting that there is a greater impact due to novel genetic changes on the makeup of the structural proteins, especially the E protein, than the non-structural proteins of these three isolates.

Several other non-unique, but uncommon, non-conservative mutations were also noteworthy. They included Capsid-T90I (polyprotein residue 90; SG/EHI-CT1372), Membrane-D40H (polyprotein residue 167; SG/EHI-MS_CT261), Envelope-I126T (residue 420 in the polyprotein; SG/EHI-MS_CT261), and NS1-Q51L (residue 845 on the polyprotein, SG/EHI-CX135 and SG/EHI-MS_CT261). Capsid-T90I was only detected in a few sequences that were reported previously from Australia (GenBank Accession Nos. AF217620, MT253735-737). Similarly, Membrane-D40H (M-D40H) was detected in a few sequences from China (GenBank Accession Nos. KT229574, KU351668, and MN544780). Envelope-I126T (E-I126T) showed a wide geographical presence in South-east Asia (Malaysia—HQ223286, Cambodia—KY927815-816, and Thailand—LC461958), while NS1-Q51L was detected in three sequences previously reported from Cambodia (GenBank Accession Nos. KY927815-816) and Thailand (GenBank Accession No. LC461958). Besides their potential phenotypic implications, these mutations also provided clues on their likely origin. In agreement with the phylogenetic analyses, these uncommon mutations suggested that SG/EHI-CT1372 was distinct and more likely to have originated from a different geographical area than SG/EHI-MS_CT261 and SG/EHI-CX135.

## 4. Discussion

All of the JEV sequences (n = 10) generated during the present study from the mosquito pools collected from 2014 to 2021 belonged to GI. This is in contrast to the detection of JEV belonging to GII locally in 2011 [36]. This observation provides further empirical evidence for the establishment of genotype I as the predominant JEV genotype in Asia [23,24,25,59]. Nine of the ten new JEV *E*-gene sequences belonged to sub-genotype GI-a, which is strongly associated with the tropics, and is often found in South-east Asia [15]. The remaining sequence (SG/EHI-CT1372) belonged to sub-genotype GI-b, which is also known as a temperate sub-genotype, and is typically found in East Asia [15].

The nine GI-a sequences formed two clusters (cluster A and cluster B, Figure 2). Both of these clusters were closely related to JEV sequences from South-east Asian countries located to the north of Singapore. This suggested that local JEV strains were likely to be introduced by migrating ardeid birds via the EAAF, which stretches from Russia and Alaska to Australia and New Zealand through East Asia and South-east Asia.

Cluster A consisted of five sequences detected in *Culex* mosquitoes within the same area during the same period (Table 2). However, no subsequent instances of JEV belonging to cluster A were observed in the following years, despite continued surveillance in the same location. This suggested that cluster A could not sustain transmission in the local ecological setting and became extinct, similar to what was observed with JEV strains “B’/B/Ishikawa type” and “C’/C/Ishikawa + Kagawa type” in Toyama, Japan by Obara, et al. [65]; this extinction could either have been due to stochastic reasons or non-favorable virus characteristics for its survival in a new environment. In contrast, the detection of cluster B four times from 2014 to 2021 may suggest its persistent presence through an enzootic transmission cycle involving the fauna in the northern and north-eastern parts of Singapore (Figure 1 and Figure 2, Table 2). Although there have been no cases of JE in Singapore for almost two decades [27], previous studies, including serological evidence of JEV infection in the local and migratory birds, and the wild boars, as well as seroconversion in sentinel chickens, have shown that JEV continues to be present in the animal populations in Singapore [26,35,36] and suggest that it may currently be enzootic in Singapore. Alternatively, cluster B JEV strains could have been imported on repeated occasions by migratory birds. These assumptions appear to support the observations that were made by Kuwata, et al. [66] on the importance of vector and/or host movement in transferring JEV strains between different regions and the maintenance of JEV strains in the local fauna within the same region over a period of time.

The detection of different variants of JEV suggested that multiple incursions of JEV have occurred in Singapore, not only over the course of this study (2014–2016 and 2019–2021), but also during the period that was studied previously [36]. Besides the phylogenetic analyses, the mutation profiling also supported this notion. For example, uncommon mutation profiles suggested that SG/EHI-CT1372 was distinct and more likely to have originated from a different geographical area than SG/EHI-MS_CT261 and SG/EHI-CX135. 

Although JEV has only been detected in the rural forested areas of Singapore thus far, increasing urbanization and the push for ecological conservation [67,68] result in close interactions between human dwellings and the habitats that are favored by culicine vectors and JEV hosts. A survey of bloodmeals of *Culex* mosquitoes in Singapore showed that JEV vectors opportunistically feed on humans [69], while a study of changing JEV epidemiological characteristics in metropolitan areas showed an apparent correlation between the presence of herons and the number of JE cases in the absence of pig farming [70]. It is thus necessary to maintain entomological and virological surveillance to keep track of possible enzootic JEV variants, thereby allowing for more leeway in containing any vector–human transmission events.

Besides gaining insights into its molecular epidemiology, the genetic characterization of JEV is also important for understanding virus fitness and virulence. Both the coding and non-coding regions of JEV are important for its replication and virulence [16,17]. While the structural and non-structural proteins provide the backbone of virion structure and replication machinery, 5′- and 3′-NCRs are also involved in genome circularization and host immune response attenuation. Genome circularization, which involves the stem loop structure SLA, is crucial for flavivirus replication, and exoribonuclease-resistant RNA (xrRNA) structures are able to mitigate the degradation of flavivirus RNA [56,71,72,73].

Among the substitutions that were detected in the study sequences (Table 6 and Table 7, and Table S5), two non-conservative substitutions E-T226A (T520A) and E-T231A (T525A) – detected in SG/EHI-CT1372 – reside within domain I, which is involved in E protein stability [74,75,76]. An E-T226R substitution was also shown to increase JEV neurovirulence and neuroinvasiveness in mice [77]; therefore, it may be useful to look into any effects of E-T226A on JEV virulence. Two E protein substitutions were also found at the neutralizing epitope position E-126 (residue 420 in the polyprotein) [76] across the study sequences in cluster A (E-I126V; Figure 2) and across the study sequences in cluster B (E-I126T; Figure 2) – these substitutions may allow for immune escape. However, no substitutions were detected in the study sequences at the E protein positions commonly found to be important for JEV neurovirulence and/or neuroinvasion (Table 6). Another important substitution was M-D40H (D167H), which was detected in SG/EHI-MS_CT261; a 40-residue region in flavivirus membrane (M) protein induces host cell apoptosis [78], and an M-I36F substitution within that region impeded JEV virion assembly in mammalian cells – likely due to the impairment of M protein apoptosis activity [79]. SG/EHI-MS_CT261 also carried an NS4b-A11T (A2283T) substitution that falls within the LIG-WD40 domain of the NS4b protein, and a mutant involving the LIG-WD40 domain has been shown to induce apoptosis and encephalitis in mouse brains [80]. Positive selection at the serine residue 24 of NS4b has been reported in genotype III of JEV that was isolated from pigs [81]. NS4b-P24S (polyprotein residue 2296) in SG/EHI-CX135 therefore warrants further investigations into its potential adaptation to swine—in particular, in the wild boar (*Sus scrofa*) as is found in Singapore. The substitution NS5-K68R (K2595R) in SG/EHI-MS_CT261 resides in the N-terminal 83-residue region of NS5, which is required for blocking IFN-α signaling [82]. However, NS5-K68R is a conservative change, and is less likely to affect the protein structure. Lastly, the most significant 3′-NCR mutations in the three whole genome sequences generated during this study were in pseudoknot 1 (PK1) of xrRNA1 (Table 5). PKs have been shown to form a mechanical block to exoribonuclease 1 by wrapping around the 5′ end of the xrRNA [83,84,85,86], hence mitigating host immune response [71,72,87]. Further research is needed to determine whether these mutations are important for adaptations to the local ecological niche.

The present study had several limitations. Firstly, the pooling of mosquitoes for screening might have confounded the detection of JEV in multiple mosquitoes of the same pool. Unfortunately, given the large number of specimens, it was not manpower- or cost-effective to screen individual mosquitoes. Secondly, the present study may not reflect the true prevalence of JE in Singapore due to the limited number of study sites, as the primary focus of this study was not to estimate the prevalence of JE in Singapore, but to determine whether there was any evidence of enzootic transmission of JEV in the country. Thirdly, although all sampling sites were selected based on the presence of ardeid birds, wild boars, water bodies, and vectors in their respective localities, vector control activities were strongly enforced in Park 2, 3, and 4 study sites (Figure 1) due to the proximity to human activity. This could affect the trapping of the mosquitoes at those sites. Lastly, the use of CDC light traps may have biased specimen collection. Lord, et al. [88] found that *Cx. tritaeniorhynchus* was oversampled with light traps in contrast to *Cx. pseudovishnui* and other potential JEV vectors and posited that this was due to the differences in the biting behaviors and host-seeking strategies rather than being indicative of the actual population density.

## 5. Conclusions

To the best of our knowledge, the present study reports the first isolation of JEV in Singapore since the abolishment of pig farms in 1992. This study is also the first record of whole genome sequences of JEV obtained from local mosquito pools. The phylogenetic analyses revealed the first description of genotype I of JEV in Singapore, as well as the possible re-establishment of sustained transmission of JEV in local fauna after its elimination in Singapore. We believe that these findings have important implications, not only for Asia and the Western Pacific, as per the recent Australian outbreak [9], but also for places outside of these regions—in particular, places where JEV is not yet enzootic/endemic but are inhabited by JEV vectors and their hosts. These areas do not necessarily need to be along the EAAF, as shown by the detection of JEV in Italy and Angola [89,90,91]. The unfolding effects of climate change tend to impact the size and the distribution of JEV vector populations, as well as vector biological properties [6,7,8], thus increasing the risk of JEV establishing transmission in such regions.

## Figures and Tables

**Table 1:** Field-caught *Culex* mosquitoes in the present study. See Table S2 for more details on the distribution of mosquitoes in the present study.

Classification	CDC Light Traps				Night Catcher Traps			
	No. of Mosquitoes Collected	No. of Mosquito Pools	No. of JEV-Positive Pools	Detection Percentage ^1^ (%)	No. of Mosquitoes Collected	No. of Mosquito Pools	No. of JEV-Positive Pools	Detection Percentage ^1^ (%)
*Culex* spp. ^2^	13,845	213	5	2.35	-	-	-	-
*Cx. bitaeniorhynchus*	33	17	0	0	32	4	0	0
*Cx. gelidus*	114	67	0	0	204	10	0	0
*Cx. quinquefasciatus*	2882	93	0	0	1	1	0	0
*Cx. sitiens*	1497	535	0	0	249	12	0	0
*Cx. tritaeniorhynchus*	14,258	926	2	0.22	10,223	291	3	1.03
*Culex* spp. *vishnui* subgroup ^3^	9646	1021	0	0	1324	71	0	0

^1^ JEV-positive pools/total pools. ^2^ From September to October 2019 only. ^3^ Excluding *Cx. tritaeniorhynchus*.

**Table 2:** Spatial and temporal information of the JEV-positive pools.

JEV Sequence ID	Location Collected	Month & Year Collected	Trap Used for Collection
SG/EHI-M_CT21_45	MSS 2	May 2021	Night Catcher trap
SG/EHI-MS_CT310	MSS 1	December 2020	Night Catcher trap
SG/EHI-MS_CT261	MSS 1	November 2020	Night Catcher trap
SG/EHI-CT1372	NR 1	November 2019	CDC light trap
SG/EHI-CX135	NR 1	October 2019	CDC light trap
SG/EHI-CX193	NR 1	October 2019	CDC light trap
SG/EHI-CX 202	NR 1	October 2019	CDC light trap
SG/EHI-CX 209	NR 1	October 2019	CDC light trap
SG/EHI-CX 214	NR 1	October 2019	CDC light trap
SG/EHI-CT1710	Park 1	June 2014	CDC light trap

**Table 3:** Percentage nucleotide and amino acid sequence similarity of the envelope gene sequences obtained from ten mosquito pools. Nucleotide sequence identities (NT ID) are in the upper right triangular area above the shaded cells and amino acid sequence identities (AA ID) are in the lower left triangular area.

	NT ID (%)	SG/EHI-CT1372	SG/EHI-CX 202	SG/EHI-CX 209	SG/EHI-CX 214	SG/EHI-CX193	SG/EHI-CX135	SG/EHI-CT1710	SG/EHI-MS_CT261	SG/EHI-MS_CT310	SG/EHI-M_CT21_45
AA ID (%)											
SG/EHI-CT1372		-	91.87	91.87	91.73	91.73	91.58	92.15	92.72	92.72	92.58
SG/EHI-CX 202		96.57	-	100	99.57	99.57	99.43	94.15	94.44	94.44	94.29
SG/EHI-CX 209		96.57	100	-	99.57	99.57	99.43	94.15	94.44	94.44	94.29
SG/EHI-CX 214		96.57	100	100	-	100	99.86	94.29	94.58	94.58	94.44
SG/EHI-CX193		96.57	100	100	100	-	99.86	94.29	94.58	94.58	94.44
SG/EHI-CX135		96.57	100	100	100	100	-	94.15	94.44	94.44	94.29
SG/EHI-CT1710		96.57	96.14	96.14	96.14	96.14	96.14	-	99.14	99.14	99
SG/EHI-MS_CT261		96.57	96.14	96.14	96.14	96.14	96.14	100	-	100	99.86
SG/EHI-MS_CT310		96.57	96.14	96.14	96.14	96.14	96.14	100	100	-	99.86
SG/EHI-M_CT21_45		96.14	95.71	95.71	95.71	95.71	95.71	99.57	99.57	99.57	-

**Table 4:** Percentage nucleotide and amino acid sequence similarity of the polyprotein sequences of three study isolates. Nucleotide sequence identities (NT ID) are in the upper right triangular area above the shaded cells and amino acid sequence identities (AA ID) are in the lower left triangular area.

	NT ID (%)	SG/EHI-CT1372	SG/EHI-CX135	SG/EHI-MS_CT261
AA ID (%)				
SG/EHI-CT1372		-	93.01	92.99
SG/EHI-CX135		95.47	-	93.33
SG/EHI-MS_CT261		94.39	96.75	-

**Table 5:** Mutations involved in significant regions of 3′-NCR secondary structures belonging to the JEV isolates in this study.

Isolate	Mutation	Region Involved
SG/EHI-CT1372	G > A	Pseudoknot 1 (PK1), found in exoribonuclease resistant RNA 1 (xrRNA1)
SG/EHI-CX135, SG/EHI-MS_CT261	T > C	
SG/EHI-CX135	C > T	3′-NCR variable region conserved sequences 1 (3′ vrCS1), found in the 3′-NCR variable region stem loop (3′vrSL)
SG/EHI-CX135	G > A	
SG/EHI-CX135, SG/EHI-MS_CT261	T > C	3′-NCR dumbbell structure conserved sequences 2 (3′dbCS2), found in the dumbbell structure 2 (DB2)

**Table 6:** Novel amino acid substitutions in the structural polyprotein of JEV genotype I. Substitutions that are only detected in the study sequences are shown. The comparison included all of the JEV GI complete coding sequences (n = 158) available in NCBI as of November 2022. Amino acid positions in the E protein that are commonly associated with JEV neurovirulence and/or neuroinvasiveness are also highlighted. Amino acid residues are numbered according to the complete polyprotein of NCBI Reference Sequence NP_059434.1. Dots indicate the residues that are identical to the consensus sequence. See Table S6 for the full list of substitutions in JEV sequences included in the table.

Protein	Capsid		prM/Membrane	Envelope												
Amino Acid Position in the Protein	36	47	63	96	107 ^‡^	123 ^‡^	138 ^‡^	176 ^‡^	177 ^‡^	226	231	264 ^‡^	279 ^‡^	360	447	492
Polyprotein Amino Acid Position	36	47	190	390	401	417	432	470	471	520	525	558	573	654	741	786
JEV GI consensus	S	V	E	F	L	S	E	I	T	T	T	Q	K	F	G	V
SG/EHI-CT1372				L						A *	A *					L
SG/EHI-CX135	G *	A												Y *	S *	
SG/EHI-CX 202	ND	ND	ND												ND	ND
SG/EHI-CX 209	ND	ND	ND												ND	ND
SG/EHI-CX 214	ND	ND	ND												ND	ND
SG/EHI-CX193	ND	ND	ND												ND	ND
SG/EHI-MS_CT261			K ^†^													I
SG/EHI-CT1710	ND	ND	ND												ND	ND
SG/EHI-MS_CT310	ND	ND	ND												ND	ND
SG/EHI-M_CT21_45	ND	ND	ND												ND	ND

ND = Not determined because only partial *E*-gene sequences (701 bp) were analyzed for respective sequences. * Non-conservative (polar/non-polar) mutation; ^†^ Conservative mutation with charge differences. ^‡^ Amino acid positions in the E protein commonly associated with JEV neurovirulence and/or neuroinvasiveness [57,58,59,60,61,62,63,64].

**Table 7:** Novel amino acid substitutions in the non-structural polyprotein of JEV genotype I. Substitutions only detected in the study sequences are shown. The comparison included all of the JEV GI complete coding sequences (n = 158) available in NCBI as of November 2022. Amino acid residues are numbered according to the complete polyprotein of NCBI Reference Sequence NP_059434.1. Dots indicate the residues that are identical to the consensus sequence. See Table S6 for the full list of substitutions in isolates included in the table and the undetermined amino acid residues of SG/EHI-CX135.

Protein	NS1			NS2A	NS3						NS4A	NS4B				NS5					
Amino Acid Position in the Protein	9	177	208	210	62	77	84	336	358	402	54	11	78	119	183	68	168	280	426	805	830
Polyprotein Amino Acid Position	803	971	1002	1356	1566	1581	1588	1840	1862	1906	2177	2283	2350	2391	2455	2595	2695	2807	2953	3332	3357
JEV GI consensus	T	D	D	S	E	I	R	L	K	D	I	A	S	T	V	K	K	R	E	K	M
SG/EHI-CT1372	I *			A *	D									I *	L					R	
SG/EHI-CX135			E	ND	ND					E				ND	ND				D		A
SG/EHI-MS_CT261		E				L	K	M	R		V	T *	A *	M *		R	R	M *			T *

ND = Not determined because of a poor quality sequence. * Non-conservative (polar/non-polar) mutations.

## Data Availability

Data and datasets supporting the conclusions of this article are provided within the article or in the Supplementary Files.

## Supplementary Materials

The following supporting information can be downloaded at: https://www.mdpi.com/article/10.3390/v14122662/s1, Figure S1: Maps of locations where (A) ardeid birds and (B) wild boars have been sighted. The maps were created with data from Wang and Hails [34] and Lamperty, Choik, Khoo, Amir, Baker, Chua, Chung, Chua, Koh, Lee, Lum, Pereira Mendes, Ngiam, O’Dempsey, Png, Sophie, Tan, Teo, Thomas, Li, Loo, Wardle and Luskin [32] respectively; Figure S2: Full JEV *E*-gene phylogenetic tree of 129 truncated JEV *E*-gene sequences; Table S1: JEV *E*-gene sequences used for the construction of the *E*-gene phylogeny tree; Table S2: Distribution of field-caught *Culex* mosquito populations; Table S3: Primers used for whole genome sequencing of JEV isolates; Table S4: JEV whole genome sequences used for the construction of the whole genome phylogeny tree; Table S5: Mutations in the present study JEV 3′-NCR secondary structures; Table S6: Full list of substitutions in JEV sequences.
