# Supplementary figures and images for: Isolation and Genetic Characterization of Japanese Encephalitis Virus Two Decades after Its Elimination in Singapore
Source: Viruses. 2022 Nov 28;14(12):2662. doi: 10.3390/v14122662 (PMC9786948; doi:10.3390/v14122662)

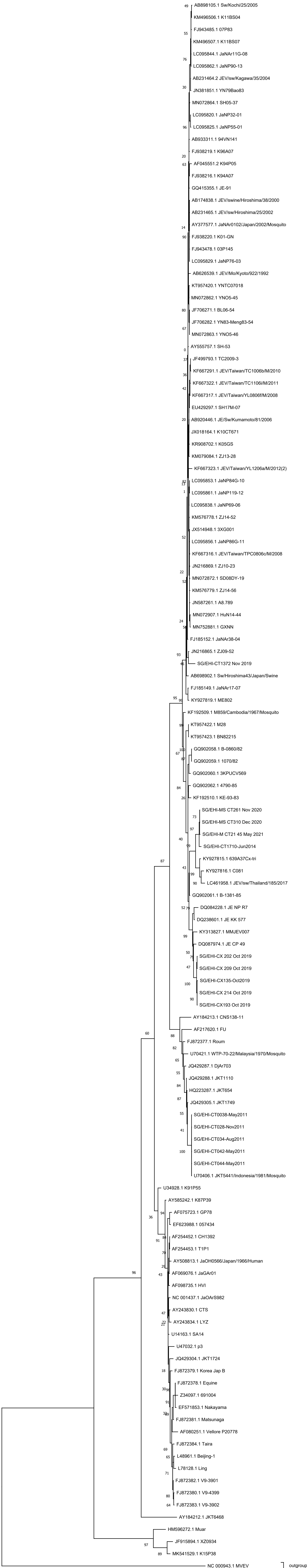

0.10

Supplement: Supplementary file 1 [file viruses-14-02662-s001.zip › Figure S1 Full JEV E-gene phylogenetic tree of 129 truncated JEV E-gene sequences.pdf]

A

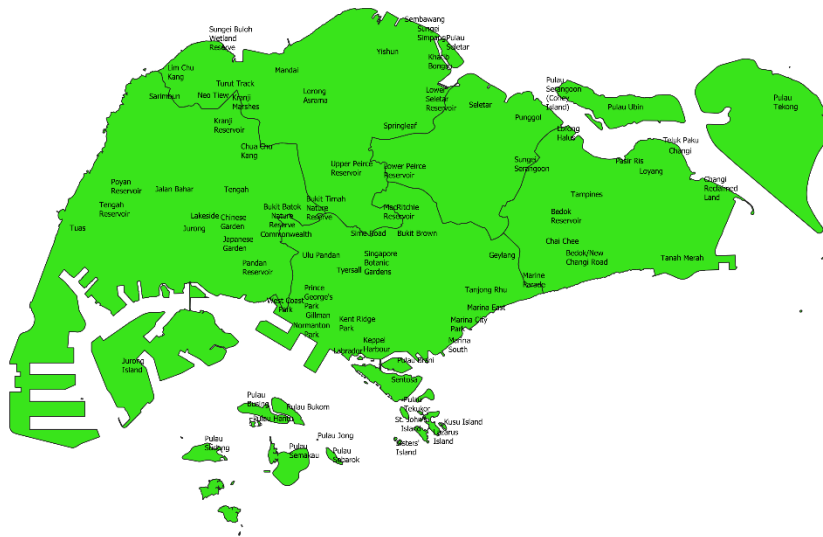

B

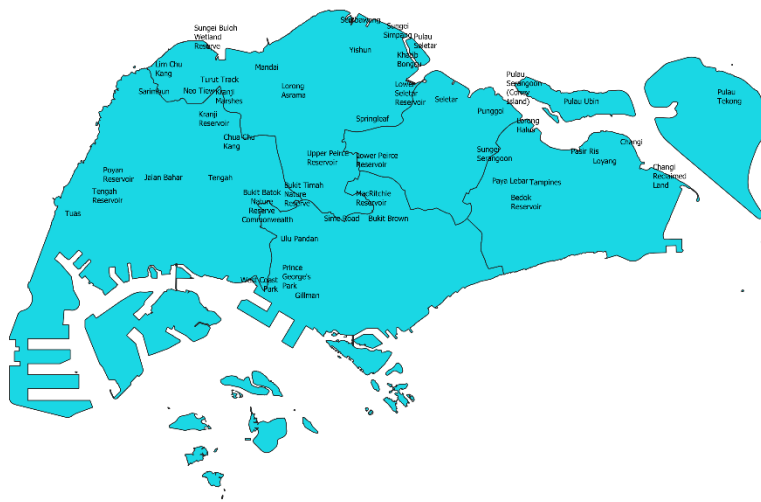

Supplement: Supplementary file 1 [file viruses-14-02662-s001.zip › Figure S2 Maps of locations where (A) ardeid birds and (B) wild boars have been sighted.pdf]
